# Nutrigenetic variants and response to diet/lifestyle intervention in obese subjects: a pilot study

**DOI:** 10.1007/s00592-021-01787-7

**Published:** 2021-09-03

**Authors:** Marica Franzago, Marta Di Nicola, Federica Fraticelli, Michele Marchioni, Liborio Stuppia, Ester Vitacolonna

**Affiliations:** 1 Department of Medicine and Aging, School of Medicine and Health Sciences, “G. D’Annunzio” University, Via dei Vestini, 66100 Chieti-Pescara, Chieti, Italy; 2 Center for Advanced Studies and Technology (CAST), “G. D’Annunzio” University, Chieti-Pescara, Chieti, Italy; 3 Laboratory of Biostatistics, Department of Medical, Oral and Biotechnological Sciences, “G. D’Annunzio” University, Chieti-Pescara, Chieti, Italy; 4 Department of Psychological, Health and Territorial Sciences, School of Medicine and Health Sciences, “G. D’Annunzio” University, Chieti-Pescara, Chieti, Italy

**Keywords:** FTO, Rs9939609, Gene–diet interaction, Nutrition, Mediterranean diet, Type 2 diabetes, Obesity, Nutrigenetics

## Abstract

**Aims:**

Nutritional and lifestyle interventions can contribute to prevent and treat obesity and its complications; however, genetic background may influence the success of a therapy. The aim of this pilot study is to evaluate the effects of the interaction between nutrigenetic variants and nutritional intervention, as well as the changes in clinical parameters and the adherence to Mediterranean diet (MedDiet) and to physical activity, of 18 overweight or obese subjects affected by T2D or dysglycemia included in a nutritional program.

**Methods:**

The subjects’ clinical parameters as well as their PREDIMED score and physical activity levels were recorded and compared at baseline, at 6 months and at the end of the intervention. Rs9939609 in *FTO*, rs17782313 near *MC4R*, rs326 in *LPL*, rs16147 in *NPY*, rs2943641 near *IRS-1* were genotyped.

**Results:**

The subjects carrying the *A* allele in *FTO* lost less weight (*p* = 0.022) and had a lower BMI decrease from baseline to 12 months (*p*-interaction = 0.047) than *TT* carriers. In addition, there was a significant PREDIMED score modification over time, according to genotypes for *FTO* rs9939609 (*p* = 0.025) and *NPY* rs16147 (*p* = 0.039), respectively.

**Conclusions:**

These preliminary findings show a significant interaction between genetic variants and the PREDIMED score, suggesting that individuals carrying the *FTO* variant may lose less weight than non-carriers through diet/lifestyle intervention.

## Introduction

Obesity is a complex and multifactorial disease with an increasing global prevalence, which has been taking on epidemic proportions over the last decades [1]. The adverse metabolic effects of overweight and obesity contribute to an increased risk of several other health conditions such as diabetes mellitus type 2 (T2D), hypertension, hyperlipidemia, and coronary heart disease [2, 3]. The pathophysiology of both obesity and obesity-related complications is complex: hereditary factors as well as socioeconomic and sociocultural milieus, have been shown to affect the risk of obesity. Some modifiable environmental factors including a sedentary lifestyle and unhealthy nutrition can cause a significant imbalance between energy intake and expenditure [4]. Moreover, due to differences in obesity susceptibility among individuals exposed to the same obesogenic environment, it has been demonstrated that genetics also plays a role in the development and the complications of this condition. In fact, the Genome-Wide Association Studies (GWAS) have identified several common single nucleotide polymorphisms (SNPs) of small impact, whose combined effects on adiposity levels, body compositions and obesity susceptibility can be captured by a polygenic risk score [5–8]. Public health strategies focus on weight management and the promotion of healthy lifestyles. Weight reduction, based on nutrition optimization and physical activity can have a beneficial effect against the overall risk and contribute to prevent and treat obesity and its complications [9]. Nevertheless, it is known that most weight loss programs are generally unsuccessful. Significantly, obese patients may be classified as normo-responders, hypo-responders, or hyper-responders, depending on their response to diet or surgical treatment [10]. This can indicate that not only physiological and behavioral factors, but also genetic background may influence the success of weight loss and the effectiveness of a shift in lifestyle behavior. In this context, Nutrigenetics is concerned with the effects of genetic variants on adverse or beneficial response to dietary components and nutrient requirements, thus identifying at-risk individuals for targeted, preventive, and early intervention strategies [11–15]. In this scenario, in order to improve the efficacy of interventions so that they became fitter to the nutritional and metabolic needs of each patient, personalized nutrition based on the metabolic profile (via clinical and biochemical assessment), the genetic profile (via nutrigenetic and nutrigenomic assessment), the body composition, and a nutritional status assessment has been suggested [16, 17]. Several studies found gene–diet interactions involving genetic variants mainly located in genes associated with obesity development, energy expenditure, adipogenesis, and eating behavior or appetite control. The most interesting issues in this field could pave the way toward personalized obesity therapy. Although the association between several SNPs and obesity/body weight has been increasingly clarified, the role of genetic risk on the effects of behavioral or environmental exposures such as diet and exercise should be better explored. Such discoveries could provide novel insight into a possible interaction among diet, genetic make-up, and other environmental factors, so as to understand the subsequent metabolic dysregulation and the effects of obesity treatment. In this light, the aim of the present pilot study is to evaluate the effects of the interaction between variants in 5 candidate genes (namely rs9939609 in *FTO*, rs17782313 near *MC4R*, rs326 in *LPL*, rs16147 in *NPY*, rs2943641 near *IRS-1*) and nutritional intervention as well as the impact/changes of the PREDIMED score and physical activity levels on the mid-term changes in the anthropometric and clinical parameters of overweight or obese subjects affected by T2D or impaired glucose regulation (IGR) over one-year period/12 months.

## Materials and methods

### Study design and participants

18 overweight or obese individuals, affected by T2D or IGR, attended by the Diabetes, Nutrition, and Metabolism Unit at “Gabriele d’Annunzio” University Hospital in Chieti, Italy, were recruited. Study protocols were approved by the Ethics Committee of the Province di Chieti and Pescara. In accordance with the Declaration of Helsinki, all participants gave their written informed consent prior to their inclusion in the study. The inclusion criteria were overweight or obese (BMI ≥ 25 and ≥ 30 kg/m^2^, respectively) subjects (male or female), ≥ 18 years of age, affected by T2D or Impaired Glucose Regulation (IGR) (Impaired Fasting Glucose or Impaired Glucose Tolerance). The exclusion criteria were the presence of Type 1 Diabetes, Eating Behavior Disorders, or other conditions that might have interfered with the development and completion of the project or cause failure to adherence to the protocol.

### Interventions and outcome assessment

All participants were included in an educational and nutritional program as described below. At baseline, each subject underwent an individual interview focusing on Medical Nutrition Therapy (MNT) and healthy lifestyle with a physician and a dietitian from the team. Demographic characteristics as well as clinical and anthropometric parameters were collected. In detail, body weight and height, Body Mass Index (BMI), waist and hip circumferences (WC and HC, respectively), HbA1c, and blood pressure were included. Moreover, adherence to the Mediterranean diet (MedDiet) was assessed using a validated 14-item questionnaire (PREDIMED) which generates a range of possible scores: no adherence (score ≤ 5), medium adherence (6 ≤ score ≤ 9), and maximum adherence (score ≥ 10) [18]. In addition, we evaluated the physical activity level using a short version of the International Physical Activity Questionnaire (IPAQ) [19].

Participants were actively involved in face-to-face individual and group-based intervention through a theoretical and practical course which included content on healthy nutrition and lifestyle.

The objectives to be achieved were, according to the MedDiet: (i) an improvement in the composition of the meals, in particular emphasizing non-starchy vegetable, whole foods over highly processed foods to the extent possible, minimizing added sugars and refined grain; (ii) a 7–10% weight decrease compared to the initial body weight achieved through an individualized eating plan; (iii) increasing moderate-intensity physical activity to at least 150 min per week [24]. The program was conducted using a nutritional and educational approach in accordance with the current literature [20–23]. The first face-to-face individual intervention was a clinical check-up and the recording of the individual’s clinical history. Also, during this first encounter, participants were instructed on how to keep a journal on dietary self-monitoring, based on the scientific literature [20–23]. The journal entries were discussed by the participant and the physician at the next meeting. To ensure the active involvement of patients in the change process, educational group sessions intervention was considered essential to improve the lifestyle of these obese and/or T2D subjects. Group-based intervention was organized in sessions performed with small groups of up to 10 people in the presence of a trained facilitator. Therefore, the endpoint of this study became the comparison between the subjects’ anthropometric and clinical parameters as well as PREDIMED and IPAQ scores at baseline, with those at 6 months (T6) and 12 months (T12) and its relationship with genetic variants.

### Gene and SNP selection

Nutrigenetic variants from five genetic loci, identified by previous GWAS or replication studies as associated with T2DM, obesity, lipid, and carbohydrate metabolism and dietary intake were selected for this study. In details, two of these variants, namely rs9939609 (*T* > *A*) in *FTO* and rs17782313 (*T* > *C*) near *MC4R*, were associated with BMI and hunger control [25–28]; rs326 (*A* > *G*) in *LPL* was involved in lipid metabolism [29–31]; rs16147 (*T* > *C*) in *NPY* was involved in adiposity and obesity [32–34]; rs2943641 (*C* > *T*) near *IRS-1* was involved in obesity, insulin resistance, T2DM risk [35].

### Genetic analysis

A blood sample from each patient was collected in a sterile tube containing EDTA and stored at + 4 °C before analysis. The genetic analysis was conducted at the Laboratory of Molecular Genetics, School of Medicine and Health Sciences, ‘‘G. d’Annunzio” University of Chieti-Pescara. Genomic DNA was automatically isolated from peripheral blood lymphocytes using MagPurix 12sAutomatedNucleicAcid Purification System (Zinexts Life Science Corp., Taiwan). Nucleic acids were quantified by measuring UV absorption using a spectrophotometer. In detail, DNA samples were amplified by PCR performed in 30 ul reaction volume containing 30 ng of genomic DNA in a Simpli-Amp™ thermal cycler (Applied Biosystems™), using the AmpliTaq Gold DNA Polymerase. Specific primers were designed on the reference gene sequence. PCR conditions were as follows: initial denaturation at 95 °C for 10 min, followed by 35 cycles of 95 °C for 30 s, 60 °C for 30 s, 72 °C for 30 s, and a final extension at 72 °C for 10 min. The amplification products were submitted to direct sequencing procedure using BigDye Term v3.1 CycleSeq Kit (Life Technologies, Monza, Italy) followed by automatic sequencing analysis (SeqStudio™ Genetic Analyzer).

### Statistical analysis

Descriptive statistics relied, after Shapiro–Wilk’s test to evaluate the departures from normality distribution for each variable, on median and interquartile range (IQR) for continuous variables and on absolute and relative frequencies (%) for qualitative variables.

Friedman test or chi-square was applied to evaluate a statistically significant variation over time for quantitative and qualitative variables, respectively.

Several linear mixed models tested the effect of different genotypes and time on features of interest; namely weight, BMI, waist-hip ratio, total cholesterol, LDH, HDL and triglyceride levels. Moreover, within linear mixed models, the interaction between genotype and time was also considered. In order to test differences in PREDIMED and IPAQ, the Friedman test was used. Three models of inheritance (i.e., dominant, recessive, and additive) were tested. If the alleles of the gene of interest are *A* and *B* in haploid, and *B* is the ‘risk’ allele, the dominant genetic model assumes that the risk is the same for heterozygotes, carrying 1 copy of the high-risk allele *B*, as for homozygotes, and the data are dichotomized into “carriers” versus “non-carriers” (i.e., ‘BB + AB’ versus ‘AA’). The recessive genetic model assumes 2 copies of *B* are required for the risk to be different from the baseline risk (i.e., ‘BB’ versus ‘AB + AA’); the additive model hypothesizes that AA, AB, and BB are associated with the lowest, the intermediate, and the highest risk, respectively (‘AA’ versus ‘AB’ versus ‘BB’) [36–38]. All tests were two-sided and a level of statistical significance was set at *p* < 0.05. Analyses were performed using the *R* software environment for statistical computing and graphics (version 4.0.5; http://www.r-project.org/).

## Results

The characteristics of 18 participants (*n* = 18, 10 males and 8 females) at baseline, at T6, and at the end of the nutritional intervention are summarized in Table 1. The median age of the participants was 64.5 (IQR: 57.5–66.8) years. Significant variations over time were observed for weight (*p* < 0.001), BMI (*p* = 0.001) and PREDIMED scores (*p* = 0.009). The median of PREDIMED score, on the scale of 0–14, was 7.5 and 8.5 at baseline and at the end of the study, respectively (*p* = 0.009).

**Table 1:** Baseline characteristics of participants

Variable	Baseline	T6	T12	*p-value*^*a*^
Weight (Kg)	96.2 (82.6,107.9)	96.5 (79.5,105.5)	91.5 (79.6, 103.2)	** < *0.001***
BMI (kg/m^2^)	31.6 (28.6, 38.0)	31.6 (28.0, 38.0)	31.1 (27.8, 36.1)	** < *0.001***
Waist circumference (cm)	114.0 (101.5, 118.8)	109.0 (97.0, 116.0)	109.0 (93.0, 116.0)	*0.264*
Hip circumference (cm)	113.5 (105.2,121.2)	115.0 (102.0,122.0)	112.0 (101.5,118.5)	*0.936*
WHR	1.0 (0.9, 1.0)	1.0 (1.0, 1.1)	1.0 (0.9, 1.0)	*0.694*
Systolic blood pressure (mmHg)	145.0 (117.5, 150.0)	132.5 (128.8, 140.0)	125.0 (120.0, 136.2)	*0.135*
Diastolic blood pressure (mmHg)	80.0 (71.2, 90.0)	80.0 (73.8, 85.0)	78.0 (70.0, 81.2)	*0.359*
PREDIMED	7.5 (7.0, 8.0)	9.0 (9.0, 10.0)	8.5 (8.0, 9.8)	***0.009***
PREDIMED_CLASS*, n(%)*				*0.761*^*b*^
- No adherence	1 (5.6%)	0 (0.0%)	0 (0.0%)	
- Adherence	14 (77.8%)	10 (58.8%)	13 (72.2%)	
- Max adherence	3 (16.7%)	7 (41.2%)	5 (27.8%)	
IPAQ*, n(%)*				*0.717*^*b*^
- low	9 (50.0%)	5 (29.4%)	4 (22.2%)	
- moderate	6 (33.3%)	8 (47.1%)	7 (38.9%)	
- high	3 (16.7%)	4 (23.5%)	7 (38.9%)	
Fasting blood glucose (mg/dl)	138.0 (115.0, 149.0)	107.0 (105.0, 108.0)	110.0 (103.5, 125.0)	*0.106*
Hba1c (%)	6.5 (6.3, 7.4)	6.2 (6.1, 6.3)	6.2 (5.4, 6.5)	*0.267*
Total cholesterol (mg/dL)	201.0 (196.0, 222.0)	208.5 (197.5, 231.8)	195.5 (164.5, 223.5)	*0.606*
HDL (mg/dL)	40.0 (37.0, 54.0)	48.5 (41.0, 54.5)	47.5 (41.8, 52.8)	*0.139*
TG (mg/dL)	139.5 (102.8, 213.8)	118.5 (94.8, 186.8)	113.0 (94.8, 145.8)	*0.078*
LDL (mg/dL)	122.4 (104.6, 142.2)	132.7 (121.9, 155.4)	124.9 (101.5, 140.2)	*0.952*

Statistically significant values are given in bold

Data are expressed as median (Q_1_–Q_3_). ^a^ *p*-value derived from Friedman Test; ^b^ *p*-value derived from chi-squared test

**Fig. 1:**
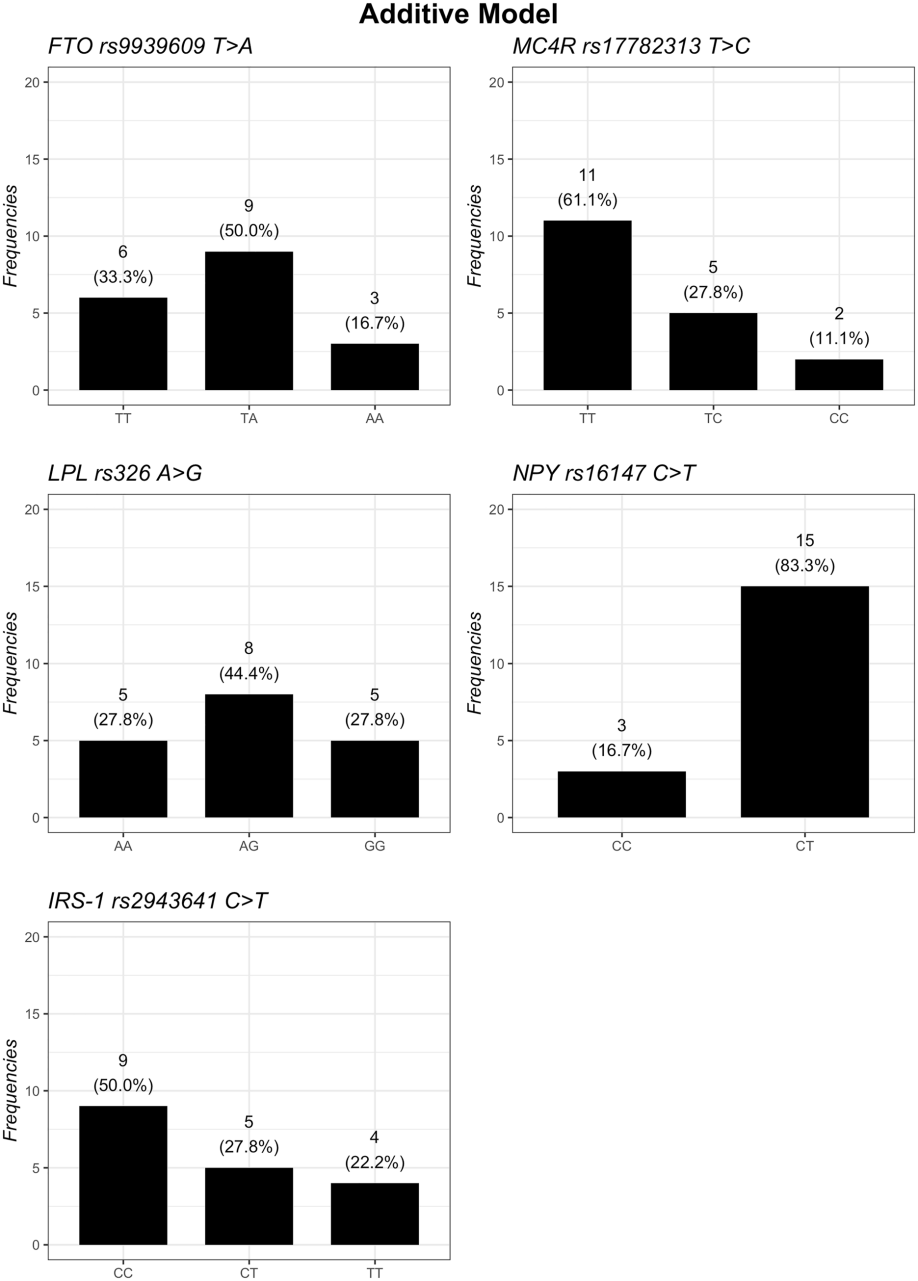
Frequencies of genetic variants according to additive genetic model

**Fig. 2:**
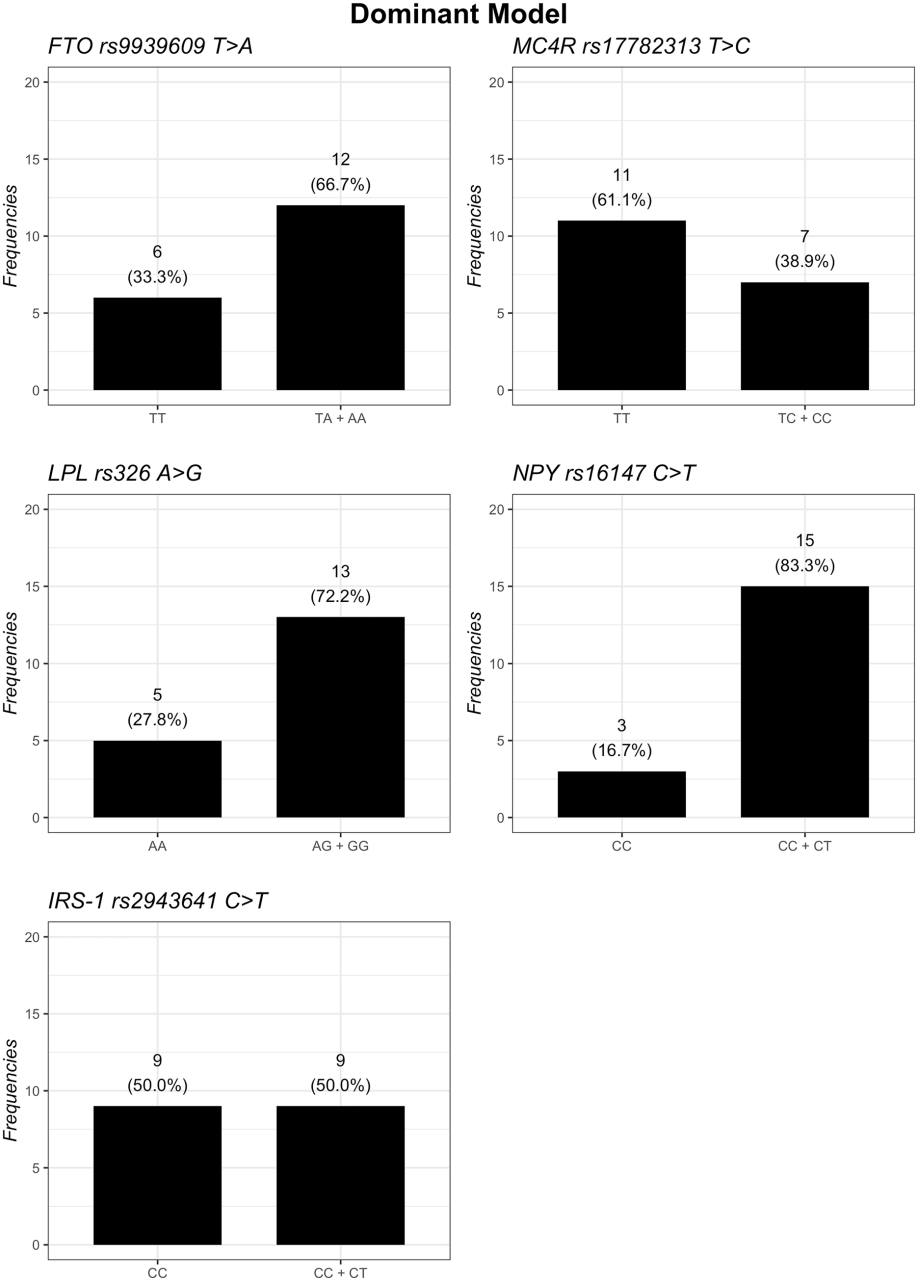
Frequencies of genetic variants according to dominant genetic model

**Fig. 3:**
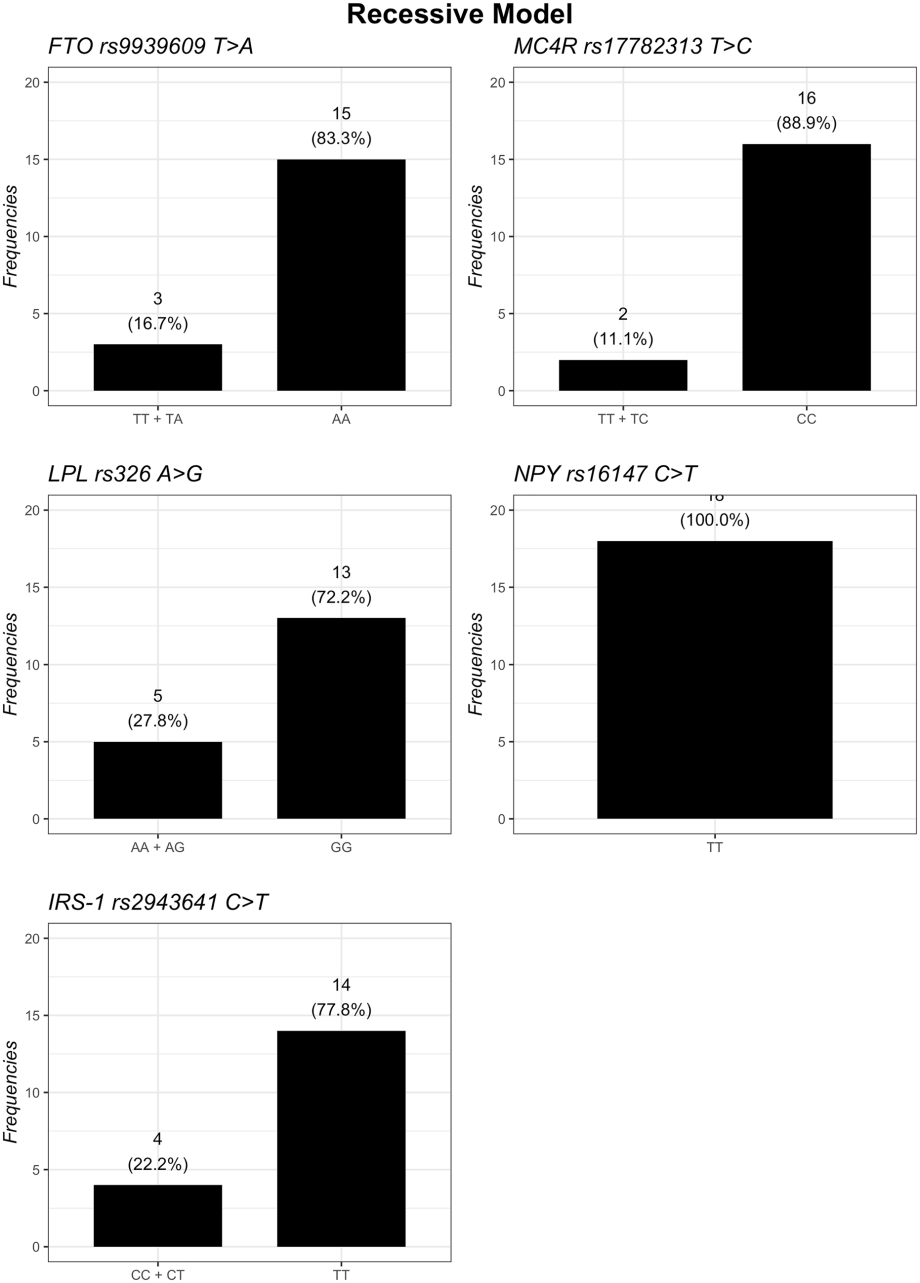
Frequencies of genetic variants according to recessive genetic model

The distribution of the genotypes based on additive, dominant, and recessive models is summarized in Figs. 1, 2 and 3. Regarding the *FTO* gene, the additive model showed that homozygous subjects for *A* risk allele had the highest baseline BMI and a statistical tendency to have lowest body weight loss after 12 months of nutritional intervention, compared to *TT* and *TA* genotypes (*p*-interaction = 0.056) (Table 2). However, the dominant model (TT vs. TA.AA) showed that subjects carrying the *A* risk allele in *FTO* lost less weight (*p*-interaction = 0.022) (Table 3) and had a lower decrease BMI from baseline to T12 (*p*-interaction = 0.047) (Table 4) than *TT* carriers. However, no difference in the recessive model (TT.TA vs AA) was found.

**Table 2:** The comparison between the subjects’ BMI at baseline with those at 6 months (T6) and 12 months (T12) and its relationship with genetic variants

				*p-value*		
*FTO* rs9939609 *T* > *A*	*TT*	*TA*	*AA*	*Genotype*^*a*^	*Time*^*b*^	*Interaction*^*c*^
Baseline T6 T12	33.3 (27.7, 38.0) 36.5 (27.8, 37.2) 31.5 (26.6, 35.8)	31.3 (28.5, 35.6) 30.6 (28.0, 35.1) 30.4 (27.8, 34.6)	36.7 (33.1, 37.7) 38.0 (33.5, 38.2) 36.1 (32.1, 37.2)	*0.907*	< *0.001*	*0.056*
*MC4R* rs17782313 *T* > *C*	*TT*	*TC*	*CC*	*Genotype*^*a*^	*Time*^*b*^	*Interaction*^*c*^
Baseline T6 T12	29.4 (28.4, 36.7) 29.5 (27.9, 36.2) 28.2 (27.0, 35.3)	31.9 (31.3, 36.7) 31.6 (30.6, 38.0) 31.9 (30.4, 36.1)	42.6 (40.3, 44.9) 41.6 (39.4, 43.7) 40.8 (38.0, 43.6)	*0.077*	< *0.001*	*0.175*
*LPL* rs326 *A* > *G*	*AA*	*AG*	*GG*	*Genotype*^*a*^	*Time*^*b*^	*Interaction*^*c*^
Baseline T6 T12	28.7 (28.2, 37.9) 32.2 (27.1, 37.6) 27.8 (26.1, 36.0)	29.6 (28.2, 35.9) 29.5 (27.3, 35.6) 29.1 (27.1, 34.7)	38.6 (31.9, 41.3) 38.3 (31.6, 40.3) 36.9 (31.9, 38.3)	*0.162*	< *0.001*	*0.966*
*NPY* rs16147 *C* > *T*	*CC*	*CT*	*TT*	*Genotype*^*a*^	*Time*^*b*^	*Interaction*^*c*^
Baseline T6 T12	28.7 (28.4, 30.3) 29.8 (28.9, 30.7) 27.8 (27.0, 29.8)	35.6 (29.0, 38.3) 35.1 (28.4, 38.2) 34.6 (28.1, 36.5)	–	*0.251*	*0.004*	*0.662*
*IRS-*1 rs2943641 *C* > *T*	*CC*	*CT*	*TT*	*Genotype*^*a*^	*Time*^*b*^	*Interaction*^*c*^
Baseline T6 T12	31.3 (28.7, 36.7) 31.1 (27.9, 37.4) 30.4 (26.1, 35.1)	35.6 (28.2, 37.9) 35.1 (28.0, 36.5) 34.6 (27.9, 36.0)	34.2 (29.4, 40.8) 34.1 (28.8, 40.2) 34.2 (28.7, 40.4)	*0.653*	< *0.001*	*0.342*

Data are expressed as median and interquartile range. Probability that the effect of nutritional intervention on BMI is influenced by:

^a^ time, for each variable, the differences have been tested between baseline, T6 and T12 of the three *Genotypes*

^b^ groups, for each variable, the differences have been tested between *Genotypes* over time

^c^ Probability that the effects of nutritional intervention is greater in one distinct group (interaction Time *genotype)

**Table 3:** The comparison between the subjects’ weight at baseline with those at 6 months (T6) and 12 months (T12) and its relationship with FTO gene according to dominant genetic model

			*p-value*		
*FTO* rs9939609 *T* > *A*	*TT*	*TA* + *AA*	*Genotype*^*a*^	*Time*^*b*^	*Interaction*^*c*^
Baseline	96.8 (82.2, 101.9)	95.0 (82.9, 112.9)	*0.720*	< *0.001*	***0.022***
T6	96.5 (79.5, 100.5)	96.2 (80.0, 111.5)			
T12	88.8 (81.5, 91.8)	94.5 (77.4, 110.4)			

Statistically significant value is given in bold

Data are expressed as median and interquartile range. Probability that the effect of nutritional intervention on weight is influenced by:

^a^ time, for each variable, the differences have been tested between baseline, T6 and T12 of the two *Genotypes*

^b^ groups, for each variable, the differences have been tested between *Genotypes* over times

^c^ Probability that the effects of nutritional intervention is greater in one distinct group (interaction Time *genotype)

**Table 4:** The comparison between the subjects’ BMI at baseline with those at 6 months (T6) and 12 months (T12) and its relationship with *FTO* gene according to dominant genetic model

			*p-value*		
*FTO* rs9939609 *T* > *A*	*TT*	*TA + AA*	*Genotype*^*a*^	*Time*^*b*^	*Interaction*^*c*^
Baseline	33.3 (27.7, 38.0)	31.6 (29.2, 37.2)	*0.945*	< *0.001*	***0.047***
T6	36.5 (27.8, 37.2)	31.1 (28.8, 38.1)			
T12	31.5 (26.6, 35.8)	31.1 (28.1, 36.7)			

Statistically significant value is given in bold

Data are expressed as median and interquartile range. Probability that the effect of nutritional intervention on BMI is influenced by:

^a^ time, for each variable, the differences have been tested between baseline, T6 and T12 of the two *Genotypes*

^b^ groups, for each variable, the differences have been tested between *Genotypes* over times

^c^ Probability that the effects of nutritional intervention is greater in one distinct group (Time *genotype)

There was a significant change in the PREDIMED score over time, according to the genotypes for *FTO* rs9939609 (*p* = 0.025) and *NPY* rs16147 (*p* = 0.039), respectively (data not shown). Regarding other participants’ characteristics relating genetic variants, no differences were detected among basal and post-treatment values of waist circumference, lipid profile, and IPAQ scores assuming a dominant, recessive and additive genetic models of inheritance (data not shown). Nevertheless, subjects with the risk-conferring *CC* genotype in *IRS-1* gene had greater decrease in total cholesterol (TC) than those without this genotype (*p-interaction* = 0.058) (Table 5) across one-year intervention, although not statistically significant.

**Table 5:** The comparison between the subjects’ total cholesterol at baseline with those at 6 months (T6) and 12 months (T12) and its relationship with genetic variants

				*p-value*		
*FTO* rs9939609 *T* > *A*	*TT*	*TA*	*AA*	*Genotype*^*a*^	*Time*^*b*^	*Interaction*^*c*^
Baseline T6 T12	212.0 (197.0, 224.8) 205.0 (196.0, 212.0) 195.5 (189.2, 202.5)	199.0 (176.5, 203.2) 199.0 (198.0, 252.0) 176.0 (159.5, 209.8)	222.0 (213.5, 234.5) 243.0 (233.0, 253.0) 243.0 (233.0, 253.0)	*0.332*	*0.859*	*0.214*
*MC4R* rs17782313 *T* > *C*	*TT*	*TC*	*CC*	*Genotype*^*a*^	*Time*^*b*^	*Interaction*^*c*^
Baseline T6 T12	207.5 (199.2, 224.8) 198.0 (195.0, 217.5) 198.0 (168.2, 218.2)	199.0 (183.0, 201.0) 257.0 (228.0, 260.0) 176.0 (164.0, 200.8)	204.0 (197.5, 210.5) 228.5 (216.8, 240.2) 221.0 (204.5, 237.5)	*0.614*	*0.669*	*0.283*
*LPL* rs326 *A* > *G*	*AA*	*AG*	*GG*	*Genotype*^*a*^	*Time*^*b*^	*Interaction*^*c*^
Baseline T6 T12	199.0 (196.0, 201.0) 225.0 (218.5, 241.0) 158.0 (151.0, 204.0)	210.0 (195.5, 226.5) 198.0 (195.0, 214.0) 195.5 (189.2, 198.0)	205.0 (199.0, 217.0) 225.5 (212.2, 238.8) 225.0 (186.0, 254.0)	*0.894*	*0.660*	*0.347*
NPY rs16147 *C* > *T*	*CC*	*CT*		*Genotype*^*a*^	*Time*^*b*^	*Interaction*^*c*^
Baseline T6 T12	199.0 (197.5, 199.0) 225.0 (225.0, 225.0) 166.0 (155.0, 185.0)	207.5 (193.2, 223.5) 205.0 (197.0, 237.5) 198.0 (186.0, 225.0)		*0.729*	*0.199*	*0.161*
*IRS-1* rs2943641 *C* > *T*	*CC*	*CT*	*TT*	*Genotype*^*a*^	*Time*^*b*^	*Interaction*^*c*^
Baseline T6 T12	201.0 (196.0, 225.0) 223.0 (202.0, 241.0) 198.5 (187.5, 223.5)	199.5 (185.8, 206.0) 203.0 (198.5, 207.5) 154.5 (149.2, 168.0)	207.5 (193.0, 211.8) 198.0 (165.5, 225.0) 226.0 (188.5, 256.2)	*0.416*	*0.609*	*0.058*

Data are expressed as median and interquartile range. Probability that the effect of nutritional intervention on total cholesterol is influenced by:

^a^ time, for each variable, the differences have been tested between baseline, T6 and T12 of the three *Genotypes*

^b^ groups, for each variable, the differences have been tested between *Genotypes* over times

^c^ Probability that the effects of nutritional intervention is greater in one distinct group (Time *genotype)

## Discussion

The main aim of the present pilot study was to assess the interaction between variants in 5 candidate genes (namely rs9939609 in *FTO*, rs17782313 near *MC4R*, rs326 in *LPL*, rs16147 in *NPY*, rs2943641 near *IRS-1*) and nutritional intervention on the changes in anthropometric and clinical parameters as well as in the PREDIMED score and the physical activity levels of overweight or obese subjects affected by T2D or IGR over one-year period/twelve months. The additive model showed that homozygous subjects for *A* risk allele in *FTO* gene presented the highest baseline BMI and had a statistical tendency to lowest body weight loss after one year of nutritional intervention, compared to *TT* and *TA* genotypes. The dominant model showed that subjects carrying the *A* risk allele in *FTO* lost less weight and had a lower BMI decrease from baseline to T12 than *TT* carriers, in spite of the same nutritional intervention. In addition, we observed a significant change in the PREDIMED score over time according to genotypes for *FTO* rs9939609. To date, the exact function of *FTO* remains undefined, but it should be noted that polymorphisms located in the *FTO* gene not only represent genetic risk factors for obesity, but have been linked with BMI, gestational diabetes mellitus (GDM), T2D, and eating behavior [39–41]. In addition, it has been suggested that the *FTO* variants may influence the expression of other genes such as the homeobox gene Iroquois-class homeobox protein 2 (IRX3) rather than the *FTO* itself [42]. Some studies showed that the *FTO* rs9939609 is associated with energy homeostasis and body composition, increasing food intake as well as appetitive behaviors reducing response in hunger and satiety after a meal [43–48]. It has also been reported that diet/lifestyle induced weight loss differs among *FTO* genotypes, although results are contradictory [49–56]. On the other hand, a systematic review and meta-analysis reported significant differences in weight loss between the *AA* and *TT* genotypes after dietary intervention [57]. Hosseini-Esfahani et al. [58] demonstrated that a higher adherence to the MedDiet was associated with lower obesity risk in subjects with more genetic predisposition to obesity, compared to those with lower MedDiet adherence and lower genetic risk score. Our results agree with more recent studies, suggesting, even in a non-significant manner, that homozygous carriers of *FTO* obesity-predisposing allele lose less weight after having followed a 4 week dietary intervention based on the Mediterranean model than non-carriers [59]. To the best of our knowledge, few and contradictory studies have assessed the effects of *LPL* rs326, *NPY* rs16147 and near *IRS-1* rs2943641 on metabolic response and weight change after a dietary intervention, thus evidence is scarce and limited. In the present study, no difference among basal and post-treatment values of anthropometric variables (BMI, weight, waist circumference, and IPAQ scores) based on genotype in *LPL, NPY* and *IRS-*1 was found. Nevertheless, subjects with the risk-conferring *CC* genotype in *IRS-1* gene had greater decreases in Total Cholesterol than those without this genotype across one-year intervention, although not statistically significant. Our finding is in line with a previous study [60], which showed that HDL cholesterol decreased, and serum triglycerides increased for each copy of the rs2943641 risk allele among T2D subjects. The insulin receptor substrate 1 (IRS1) is considered a major mediator between the insulin receptor and phosphatidylinositol 3-kinase (PI3K) in the insulin signaling pathway and it has been reported that rs2943641, located in inter-genic region 500 kb upstream from the *IRS1* may have an effect on IRS1 expression associated PI3K activity [61, 62]. Subjects with *CC* genotype of the rs2943641 showed a higher improvement in insulin resistance than *T* allele carriers when adhering to a high-carbohydrate and low-fat diet in a 2 year randomized trial [63]. Thus, although the mechanisms underlying the interaction between the *IRS1* gene variant and diet on insulin action remain to be elucidated, the activation of IRS1 associated PI3K activity may be enhanced in rs2943641 *CC* subjects in response to a low-fat and high-carbohydrate diet. In our study, we showed a significant change of the PREDIMED score from baseline to month 12 according to genotypes for *NPY* rs16147. NPY is an orexigenic neuropeptide that regulates the hypothalamic control of energy, immune function, and cardiovascular function [64–66]. Variants in the *NPY* gene have been associated with several human diseases stimulating food intake, decreasing energy expenditure, and increasing energy stored [67]. In this view, lymphoblastoid cell lines showed higher NPY expression in risk allele carriers of rs16147, demonstrating allele-specific effects on *NPY* gene expression and NPY peptide concentrations [68, 69]. Regarding body weight regulation, it has been showed that the rs16147 variant affected anthropometric, biochemical and inflammatory parameters in response to dietary interventions in overweight or obese subjects [70, 71], and its effect on central obesity and abdominal fat distribution were modified by dietary fat, suggesting that individuals carrying the *C* allele of the *NPY* rs16147 SNP might benefit more by taking a high-fat weight loss diet [72]. Recently, Martin et al. [73] demonstrated that the *A* allele of this *NPY* variant produces a better metabolic response in terms of insulin resistance and basal insulin secondary to weight loss with two different hypocaloric diets in obese subjects, with improvement being higher with the Mediterranean diet. Further studies are needed to elucidate whether genetic variants may influence the outcomes of the nutritional intervention. To date, it should be emphasized that there are discrepancies in previous weight loss clinical studies due to: (i) the types of interventions, including energy-restricted diets, duration of the intervention, and physical activity interventions; (ii) the participants’ heterogeneity in what concerns age (adults, older adults), ancestry, nutritional status (overweight, obese, severely obese), pathologic situation/status (prediabetic, diabetic); (iii) the dietary assessment method that can produce inaccurate dietary data collection [74]. Therefore, with regard to the analysis of gene–diet interactions, all the aspects mentioned above make comparisons between findings difficult and should be considered in order to achieve comparability across different studies. The strength of our study is that it is based on intensive nutritional intervention. However, several limitations of this pilot study warrant consideration. One limitation is that the sample size of the current study is small for a genetic association study. In addition, although personalized nutrition refers mainly to a person’s genetic background, the composition of gut microbiota and epigenetic markers may modify gene expression and could be involved in the outcome of weight loss interventions. In the era of precision medicine, the integration of nutrigenetic, epigenetic, as well as metagenomic data may offer meaningful opportunities for the design of more personalized dietary treatments to optimize an individual’s response to dietary interventions, as well as to improve the prevention and treatment of metabolic disturbances, such as T2M, GDM, hypertension, and cardiovascular diseases [13, 15]. In summary, our study showed an interaction between the modification of the PREDIMED score over time and *FTO* as well as *NPY*. Although it remains unknown whether genetic risk for obesity is associated with timing of food choices [75], genetic variants with central nervous system functions could affect the control of food intake and food-choice behavior. Future clinical intervention studies should explore whether food choices may influence the causal pathway between genetic risk and weight gain. In addition, our preliminary findings support the impact of the interaction between *FTO* gene and diet/lifestyle intervention in the regulation of body weight. Even though the suboptimal statistical power resulting from the small sample size prevents us from clearly demonstrating the effect of the genetic variants on nutritional intervention, we believe that further studies on the role of *FTO*, a key gene in food intake and appetite, are highly needed with regard to lifestyle intervention. Therefore, an extension of our preliminary findings in larger, multicenter cohorts is warranted.

If *FTO* rs9939609 is confirmed to influence the decreasing of BMI and body composition during nutritional treatments, this notion can encourage a shift toward both the development of personalized nutritional advice and monitoring based on joint associations of dietary pattern and genotypes. It should be noted that favorable lifestyle should be universally recommended in obesity prevention, regardless of genetic make-up, thus supporting current public health guidelines.

## Conclusions

In summary, our pilot study showed an interaction between the modification of the PREDIMED score over time and *FTO* as well as *NPY*. In addition, our findings support the interaction between *FTO* gene and diet/lifestyle intervention in the regulation of body weight. So, we can say that if *FTO* rs9939609 is confirmed to influence the decreasing of BMI and body composition during nutritional treatments, this can encourage a shift toward both the development of personalized nutritional advice and monitoring based on joint associations of dietary pattern and genotypes.

## Data Availability

The data underlying this article will be shared on reasonable request to the corresponding author.
